# Ovarian imaging radiomics quality score assessment: an EuSoMII radiomics auditing group initiative

**DOI:** 10.1007/s00330-022-09180-w

**Published:** 2022-10-27

**Authors:** Andrea Ponsiglione, Arnaldo Stanzione, Gaia Spadarella, Agah Baran, Luca Alessandro Cappellini, Kevin Groot Lipman, Peter Van Ooijen, Renato Cuocolo

**Affiliations:** 1 Department of Advanced Biomedical Sciences, University of Naples Federico II, Naples, Italy; 2 Department of Diagnostic and Interventional Radiology, University of Cologne, Cologne, Germany; 3 Department of Biomedical Sciences, Humanitas University, Pieve Emanuele, Milan, Italy; 4 Department of Radiology, Netherlands Cancer Institute, Amsterdam, Netherlands; 5 Department of Radiation Oncology, University Medical Center Groningen, Groningen, The Netherlands; 6 Machine Learning Lab, Data Science Center in Health, University Medical Center Groningen, Groningen, the Netherlands; 7 Department of Medicine, Surgery and Dentistry, University of Salerno, Baronissi, Italy; 8 Augmented Reality for Health Monitoring Laboratory (ARHeMLab), Department of Electrical Engineering and Information Technology, University of Naples “Federico II”, Naples, Italy

**Keywords:** Machine learning, Ovary, Computed tomography, Magnetic resonance imaging, Positron emission tomography

## Abstract

**Objective:**

To evaluate the methodological rigor of radiomics-based studies using noninvasive imaging in ovarian setting.

**Methods:**

Multiple medical literature archives (PubMed, Web of Science, and Scopus) were searched to retrieve original studies focused on computed tomography (CT), magnetic resonance imaging (MRI), ultrasound (US), or positron emission tomography (PET) radiomics for ovarian disorders’ assessment. Two researchers in consensus evaluated each investigation using the radiomics quality score (RQS). Subgroup analyses were performed to assess whether the total RQS varied according to first author category, study aim and topic, imaging modality, and journal quartile.

**Results:**

From a total of 531 items, 63 investigations were finally included in the analysis. The studies were greatly focused (94%) on the field of oncology, with CT representing the most used imaging technique (41%). Overall, the papers achieved a median total RQS 6 (IQR, −0.5 to 11), corresponding to a percentage of 16.7% of the maximum score (IQR, 0–30.6%). The scoring was low especially due to the lack of prospective design and formal validation of the results. At subgroup analysis, the 4 studies not focused on oncological topic showed significantly lower quality scores than the others.

**Conclusions:**

The overall methodological rigor of radiomics studies in the ovarian field is still not ideal, limiting the reproducibility of results and potential translation to clinical setting. More efforts towards a standardized methodology in the workflow are needed to allow radiomics to become a viable tool for clinical decision-making.

**Key Points:**

*• The 63 included studies using noninvasive imaging for ovarian applications were mostly focused on oncologic topic (94%).*

*• The included investigations achieved a median total RQS 6 (IQR, −0.5 to 11), indicating poor methodological rigor.*

*• The RQS was low especially due to the lack of prospective design and formal validation of the results.*

**Supplementary Information:**

The online version contains supplementary material available at 10.1007/s00330-022-09180-w.

## Introduction

Radiomics represent a new comprehensive research field combining quantitative image analysis, artificial intelligence, and medical imaging [1]. This discipline allows for the extraction of information from imaging data that could not be detectable by the human eye [2, 3]. Such data may be used to create classification models able to provide diagnostic and prognostic outputs and serve as decision-support tools [4, 5]. Several studies applied radiomics to the field of ovarian imaging, being especially focused on oncologic patients [6–8]. As a matter of fact, in the last decade, there has been an increasing clinical demand for improvements in diagnostic accuracy and patient risk stratification. In this light, predictors extracted by noninvasive imaging techniques could be worthy in several clinical scenarios, such as for classifying ovarian masses or predicting their clinical outcome [9–11]. However, radiomics applications still remain confined to academic research due to the intrinsic complexity of the method and the limited reproducibility of the numerous processes involved, especially regarding image segmentation, feature extraction, and dataset analysis [12]. Therefore, a standardized assessment of the accuracy, reproducibility as well as the clinical utility of radiomics data is needed. Aiming to respond to these demands, Lambin et al proposed the radiomics quality score (RQS), a system of metrics for the overall evaluation of the methodological validity and thoroughness of radiomics-based studies [13]. This tool has been already adopted to assess the scientific rigor of radiomics-based studies in different topics, mainly focused on oncology, such as prostate, renal, and breast cancer [14–16]. In the last years, together with the increasing clinical demand for non-invasive diagnostic techniques in the ovarian field, we have been experiencing an ever-growing number of scientific research extracting features from medical images, aimed at tumor detection and characterization or to predict prognosis and response to therapy [10, 17, 18].

Therefore, the aim of our systematic review was to evaluate the methodological rigor of investigations using computed tomography (CT), magnetic resonance imaging (MRI), positron emission tomography (PET), or ultrasound (US) for ovarian assessment on which radiomics-based models for diagnostic or prognostic purposes have been explored.

## Methods

### Protocol and registry

This study followed the PRISMA (Preferred Reporting Items for Systematic Reviews and Meta-Analyses) statement [19]. The review protocol is registered on PROSPERO (CRD42021293541).

### Search strategy

An English literature search was performed in consensus by two investigators (A.P. and A.S.) using the PubMed, Scopus, and Web of Science databases to identify articles published up to November 19^th^, 2021. The following search terms and their variations were used: “radiomics” AND “ovary” AND “computed tomography” OR “magnetic resonance” OR “positron emission tomography” OR “ultrasound”. The detailed search string is available in the supplementary materials. After the removal of duplicates, all abstracts were assessed to remove papers other than original research (e.g., reviews, editorials, case reports), investigations not focused on the topic of interest, or not involving human subjects.

### Data collection and study evaluation

The RQS was used to evaluate the methodological rigor of included papers [13]. It consists of 16 items regarding different steps in the workflow of radiomics. The summed total score ranges between −8 and 36, while the percentage is calculated on a 0–36 scale (Table 1). Two readers with previous experience in radiomics and the RQS (A.P. and G.S.) evaluated the papers in consensus. Disagreements were resolved by a third reviewer (R.C.), who reviewed the controversial items after reading the corresponding full text and discussed them with the other readers to reach a consensus. The full manuscripts were assessed to collect the following data: first author category (medical or other), study aim (diagnostic or prognostic), topic (oncology or other) and design (single-center or multi-center), imaging modality (CT, MRI, PET or US), journal quartile (first or other, based on Scopus data), segmentation strategy, machine learning algorithm, and total number of included patients.

**Table 1:** Overview of radiomics quality score items and mode of the corresponding scores in the included papers

RQS checkpoint	RQS item number and name	Description and (points)	Mode
First	Item 1: Image protocol quality	Well-documented protocol (+1) AND/OR publicly available protocol (+1)	1
Second	Item 2: Multiple segmentation	Testing feature robustness to segmentation variability: e.g. different physicians/algorithms/software (+1)	0
	Item 3: Phantom study	Testing feature robustness to scanner variability: e.g. phantom studies/different vendors/scanners (+1)	0
	Item 4: Multiple time points	Testing feature robustness to temporal variability: e.g. organ movement/expansion/shrinkage (+1)	0
Third	Item 5: Feature reduction	Either feature reduction OR adjustment for multiple testing is implemented (+3); otherwise (-3)	3
	Item 6: Multivariable analysis	Non-radiomics feature are included in/considered for model building (+1)	0
	Item 7: Biological correlates	Detecting and discussing the correlation of biology and radiomics features (+1)	0
	Item 8: Cut-off analysis	Determining risk groups by either median, pre-defined cut-off, or continuous risk variable (+1)	0
	Item 9: Discrimination statistics	Discrimination statistics and its statistical significance are reported (+1); a resampling technique is also applied (+1)	1
	Item 10: Calibration statistics	Calibration statistics and its statistical significance are reported (+1); a resampling technique is also applied (+1)	0
	Item 11: Prospective design	Prospective validation of a radiomics signature in an appropriate trial (+7)	0
	Item 12: Validation	Validation is missing (-5) OR internal validation (+2) OR external validation on a single dataset from one institute (+3) OR external validation on two datasets from two distinct institutes (+4) OR validation of a previously published signature (+4) validation is based on three or more datasets from distinct institutes (+5)	-5
	Item 13: Comparison to “gold standard”	Evaluating model’s agreement with/superiority to the current “gold standard” (+2)	2
	Item 14: Potential clinical application	Discussing model applicability in a clinical setting (+2)	0
	Item 15: Cost-effectiveness analysis	Performing the cost-effectiveness of the clinical application (+1)	0
	Item 16: Open science and data	Open-source scans (+1) AND/OR open-source segmentations (+1) AND/OR open-source code (+1) AND/OR open-source representative features and segmentations (+1)	0

*RQS* indicates radiomics quality score [13]

### Statistical analysis

The Shapiro-Wilk test was performed to evaluate the normality of distribution for continuous variables. These are presented as median and interquartile range (IQR) whereas categorical data are as counts and percentages. Subgroup analyses were performed to establish whether the total RQS varied according to first author category, study aim, topic, imaging modality, and journal quartile, using the Mann-Whitney U test or Kruskal-Wallis rank test. When a paper belonged to more than one category it was counted for each within the sub-analysis. Statistical analyses were performed with the “stats” (v4.1.3) R package (v4.1.3) [20]. A *p* value < 0.05 was considered statistically significant.

## Results

### Literature search

**Fig. 1:**
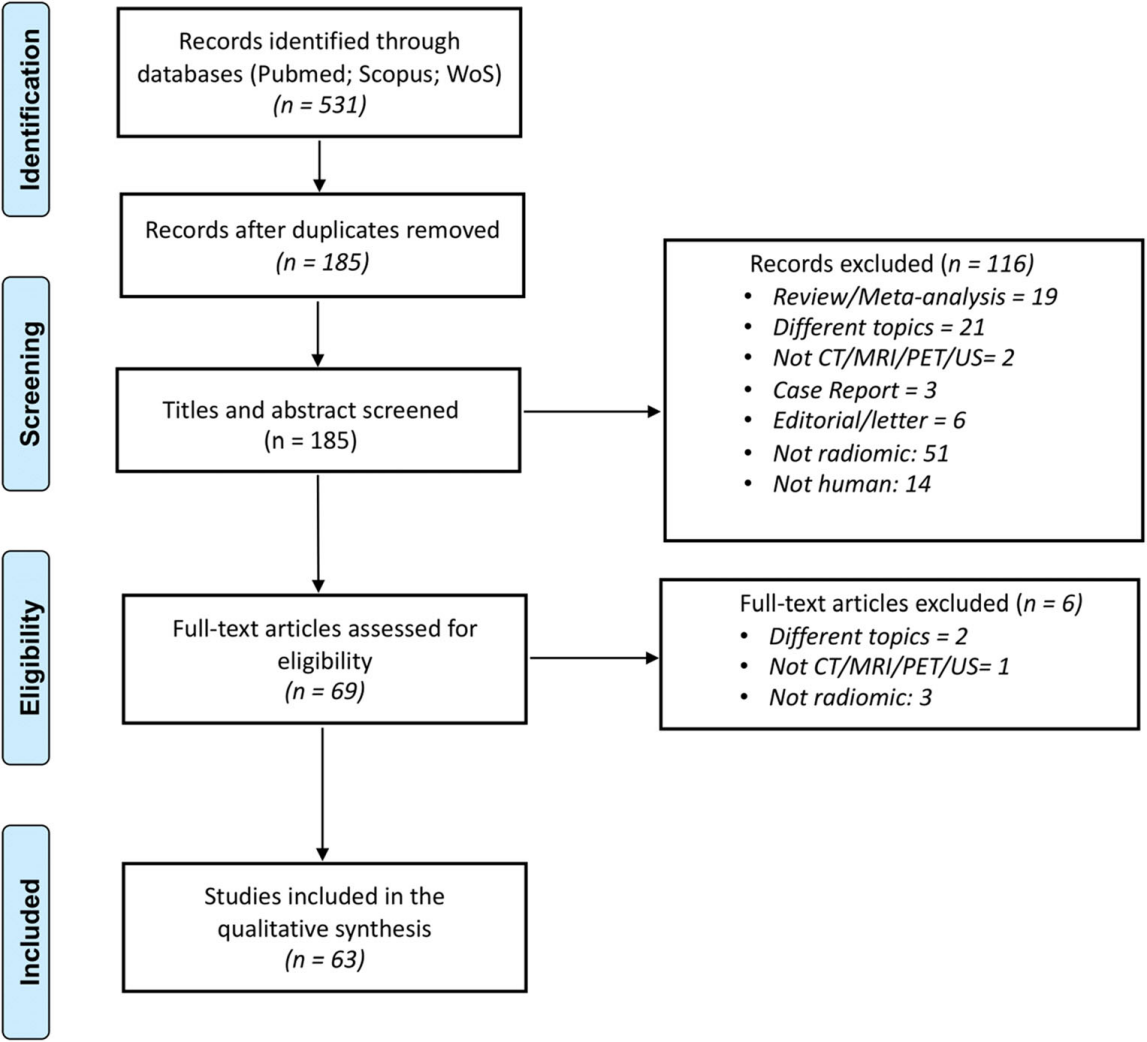
Literature search and study selection flowchart

The study selection flowchart is shown in Fig. 1. The initial search identified 531 potentially eligible articles, 346 of which were duplicates. The reviewers, after the evaluation of the titles and abstracts of the remaining 185 papers studies removed 116 citations. Then, investigators blindly reviewed the full text of the remaining 69 articles, and 6 of these were excluded. Finally, 63 papers were included in the systematic review.

### Study characteristics

**Fig. 2:**
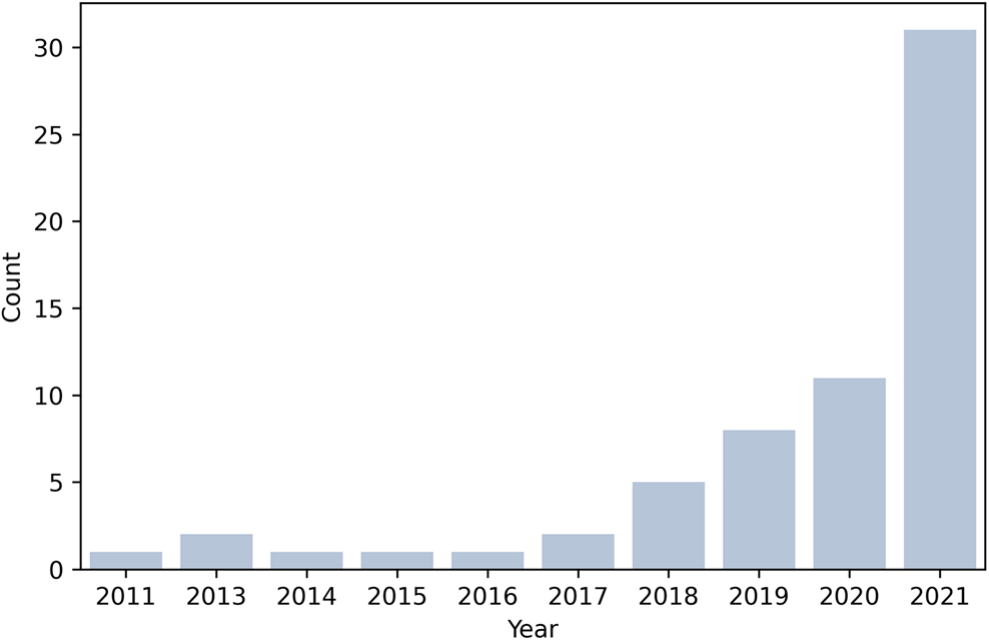
Count plot showing the number of CT, MRI, PET, and US radiomics studies in ovarian setting published over the years

The characteristics of the included studies are shown in the supplementary Table 1. The median population number was 105 (IQR, 67–217). Among the included papers, 49% (31/63) were published in 2021, 17% (11/63) in 2020, 13% (8/63) in 2019, 8% (5/63) in 2018, 3% (respectively 2/63 per year) in 2013 and 2017, and 2% (respectively 1/63 per year) in 2011, 2014, 2015, and 2016 (Fig. 2). The first author of most of the investigations (78%, 14/63) was a medical doctor. Radiomics analysis was conducted with diagnostic and prognostic aims respectively in 68% (43/63) and 30% (19/63) of the studies, whereas in 2% (1/63) of the investigations it was used with both intended purposes. CT was the most used imaging technique (41%, 26/63). MRI and US were respectively adopted in 34% (22/63) and 22% (14/63) of the studies, whereas in 2% (1/63) of the investigations both PET and CT were used. As for the segmentation method, regions of interest were largely annotated manually on medical images (78%, 49/63), being three-dimensional in most cases (70%, 44/63). Finally, regarding machine learning algorithms, a high heterogeneity was found, with a minority of works adopting deep learning strategies (8%, 5/63) and the most embraced approach being overall logistic regression (40%, 25/63).

### Study evaluation

**Fig. 3:**
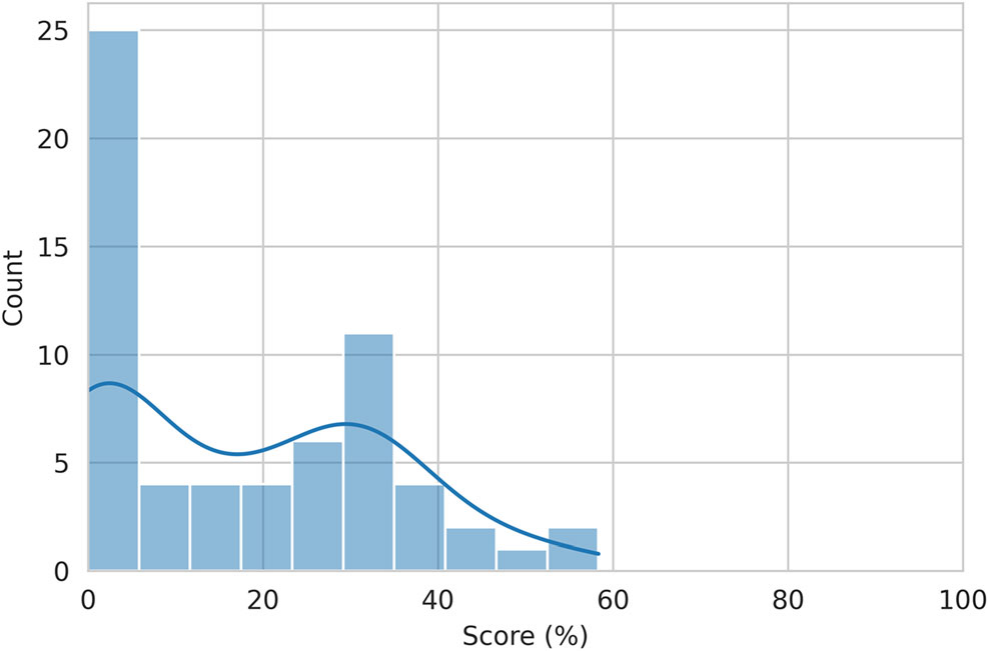
Distribution of median total RQS percentage score of investigations included in our review. This is presented both as a histogram (bars) and its corresponding density function (line)

**Fig. 4:**
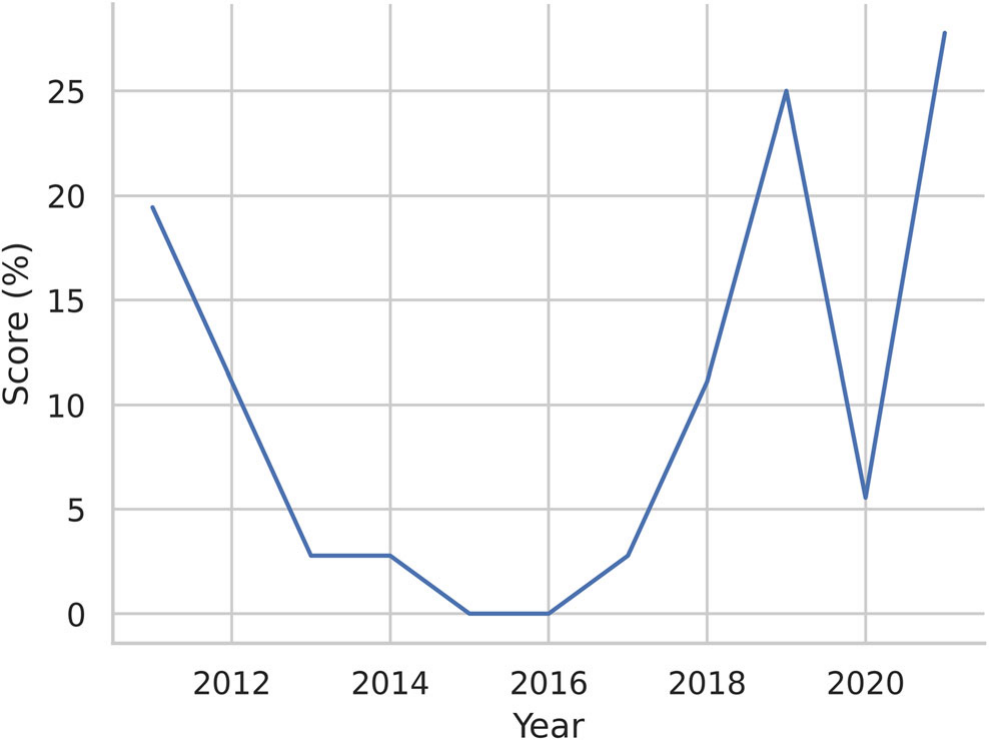
Line plot of median total RQS percentage in relation to the publication year

Results are detailed in Table 2. Overall, the 63 included investigations obtained a median total RQS of 6 (IQR, −0.5 to 11), corresponding to a percentage of 16.7% (IQR, 0–30.6%) (Fig. 3). Median RQS distribution over the years is shown in Fig. 4. In regard of the first RQS checkpoint, the Authors included comprehensive information of their imaging protocol in 71% (45/63) of the corresponding investigations. In the second RQS checkpoint, features robustness to segmentation variability was assessed in 29% of the papers (18/63), while only one study (2%) performed a phantom experiment. Concerning the third RQS checkpoint, 76% (48/63) of the studies used reduction techniques to avoid feature overfitting, while less than half of the investigations (29/63) included non-radiomics features for model building. Discrimination statistics were usually performed (86%, 59/63), while only 6% (4/63) of the investigations had a prospective design. Validation, either internal or external, of the results was missing in about half of the included studies (51%, 32/63). A direct comparison between radiomics and the current gold standard was performed in 52% (33/63) of the investigations, whereas 24% (15/63) of the papers presented a formal assessment of radiomics models’ clinical utility. Finally, only one study (2%) performed a cost-effectiveness analysis and 8 studies (13%) made their code and/or data publicly available.

**Table 2:** Radiomics quality scores for all included studies

Author (year)	Item 1	Item 2	Item 3	Item 4	Item 5	Item 6	Item 7	Item 8	Item 9	Item 10	Item 11	Item 12	Item 13	Item 14	Item 15	Item 16	RQS (total)	RQS (%)
Acharya (2013)	1	0	0	0	-3	0	0	0	0	0	0	2	0	2	0	0	2	5,6
Acharya (2014)	0	0	0	0	3	0	0	0	1	0	0	-5	2	0	0	0	1	2,8
Ai (2021)	1	0	0	0	3	1	0	1	2	0	0	2	2	0	0	0	12	33,3
Al-Karawi (2021)	0	0	0	0	-3	0	0	0	2	0	0	-5	2	2	0	0	-2	0
An (2021)	1	1	0	1	3	1	0	0	1	0	0	-5	2	0	0	0	5	13,9
Beer (2020)	1	1	0	0	-3	1	1	1	1	0	0	-5	0	0	0	1	-1	0
Chen (2021)	1	1	0	1	3	1	0	0	2	1	0	2	2	2	0	0	16	44,4
Chen (2021)	1	1	0	0	3	0	0	0	2	1	0	2	2	2	0	0	14	38,9
Chiappa (2021)	0	1	0	1	3	0	0	0	0	0	0	2	2	0	0	0	9	25
Chiappa (2021)	0	0	0	0	-3	1	0	0	1	0	0	-5	2	2	0	1	-1	0
Danala (2017)	1	0	0	0	3	0	0	0	1	0	0	-5	2	0	0	0	2	5,6
Faschingbauer (2013)	0	0	0	0	-3	0	0	0	2	0	0	-5	0	0	0	0	-6	0
Fathi Kazerooni (2018)	1	0	0	0	3	0	0	0	0	0	7	-5	0	0	0	0	6	16,7
He (2020)	1	1	0	0	-3	0	0	0	1	0	0	-5	0	0	0	0	-5	0
Himoto (2019)	1	0	0	0	-3	1	0	0	2	0	0	-5	0	0	0	1	-3	0
Hu (2021)	1	0	0	0	3	1	0	0	2	1	0	2	0	0	0	0	10	27,8
Jian (2021)	0	0	0	0	3	0	1	1	0	0	0	5	0	0	0	0	10	27,8
Khazendar (2015)	0	0	0	0	-3	0	0	0	2	0	0	-5	2	0	0	0	-4	0
Kiruthika (2018)	0	0	0	0	3	0	0	0	1	0	0	-5	0	0	0	0	-1	0
Kyriazi (2011)	1	1	0	1	-3	1	1	0	1	0	7	-5	2	0	0	0	7	19,4
Lee (2021)	0	0	0	0	-3	0	0	0	0	0	0	-5	0	0	0	0	-8	0
Li H (2021)	0	0	0	0	3	1	0	1	1	0	0	2	0	0	0	0	8	22,2
Li HM (2019)	1	0	0	0	-3	0	1	0	1	0	0	-5	0	0	0	1	-4	0
Li HM (2020)	1	1	0	0	-3	0	1	0	1	0	0	-5	0	0	0	0	-4	0
Li HM (2021)	1	0	0	0	3	1	0	0	1	0	0	-5	2	0	0	0	3	8,3
Li MR (2021)	1	0	0	0	3	0	0	0	1	0	0	2	2	0	0	0	9	25
Li NY (2021)	1	1	0	0	3	0	0	0	1	0	0	-5	2	0	0	1	4	11,1
Li S (2021)	1	0	0	0	3	1	0	0	1	1	0	3	2	2	0	0	14	38,9
Li YA (2020)	1	1	0	0	3	0	0	0	1	0	0	4	2	0	0	0	12	33,3
Lu H (2019)	0	0	0	0	3	1	1	0	0	0	0	3	2	0	0	1	11	30,6
Lu J (2021)	1	0	0	1	-3	0	0	1	1	0	0	-5	2	0	0	0	-2	0
Lupean (2020)	0	0	0	0	3	1	0	0	1	0	0	-5	0	0	0	0	0	0
Lupean (2020)	1	0	0	0	-3	0	0	1	1	0	0	-5	0	0	0	0	-5	0
Meier (2019)	1	0	0	0	3	0	0	0	0	0	0	-5	0	0	0	0	-1	0
Mimura (2016)	1	0	0	0	-3	0	0	0	1	0	0	-5	2	0	0	0	-4	0
Nero C (2020)	0	0	0	0	3	0	0	0	2	0	0	2	2	0	0	0	9	25
Pan (2020)	1	0	0	0	3	1	0	0	1	1	0	3	0	2	0	0	12	33,3
Park H (2021)	1	0	0	0	3	1	0	0	1	0	0	-5	0	0	0	0	1	2,8
Qi (2021)	0	0	0	0	3	1	0	0	1	1	0	2	2	2	0	0	12	33,3
Qian (2020)	1	1	0	0	3	1	1	0	2	2	0	2	2	2	0	0	17	47,2
Rizzo (2018)	1	0	1	0	3	1	0	0	1	0	0	-5	2	0	0	0	4	11,1
Seo (2021)	1	1	0	0	3	0	0	0	0	0	0	-5	0	0	0	0	0	0
Song (2021)	1	0	0	0	3	1	0	0	2	1	7	2	0	2	0	0	19	52,8
Song (2021)	1	1	0	0	3	0	0	0	1	1	7	2	2	2	0	1	21	58,3
Stefan (2021)	1	1	0	0	3	0	0	1	1	0	0	-5	0	0	0	0	2	5,6
Ștefan (2021)	1	1	0	1	3	0	1	1	1	0	0	-5	2	0	0	0	6	16,7
Vargas (2017)	0	0	0	0	3	0	0	0	0	0	0	-5	0	0	0	0	-2	0
Veeraraghavan (2020)	0	0	0	0	3	0	1	0	1	0	0	-5	2	0	0	0	2	5,6
Wang R (2021)	1	1	0	0	3	1	0	0	2	0	0	2	0	0	0	1	11	30,6
Wang S (2019)	1	0	0	0	3	1	0	0	1	0	0	3	2	0	0	0	11	30,6
Wang X (2021)	1	0	0	0	3	1	0	0	2	1	0	2	0	0	0	0	10	27,8
Wei C (2020)	1	0	0	0	3	1	0	0	2	0	0	-5	2	0	0	0	4	11,1
Wei W (2018)	0	0	0	0	3	0	0	0	1	0	0	2	0	0	0	0	6	16,7
Wei W (2019)	1	0	0	0	3	1	0	0	2	1	0	3	2	0	0	0	13	36,1
Yao (2021)	1	1	0	0	3	1	0	1	1	0	0	2	2	0	0	0	12	33,3
Ye (2021)	1	0	0	0	3	1	1	0	1	0	0	2	2	0	0	0	11	30,6
Yi (2021)	1	0	0	0	3	1	0	0	1	1	0	2	0	2	0	0	11	30,6
Xu XP (2021)	1	1	0	0	3	0	0	0	1	0	0	2	0	0	0	0	8	22,2
Yu XY (2021)	1	0	0	0	3	1	0	0	1	1	0	2	0	2	0	0	11	30,6
Zargari (2018)	1	0	0	0	3	0	0	0	2	0	0	-5	0	0	0	0	1	2,8
Zhang H (2019)	1	0	0	1	3	0	0	1	1	0	0	2	2	2	0	0	13	36,1
Zhang L (2019)	0	0	0	0	3	0	0	0	1	0	0	2	0	0	1	0	7	19,4
Zhu (2021)	1	0	0	0	3	1	0	1	2	2	0	2	2	2	0	0	16	44,4

The total score ranges from −8 to 36 and the percentage was based on the maximum value of 36

*RQS* indicates radiomics quality score [13]

### Subgroup analysis

Table 3 shows the results of the subgroup analysis according to first author category, study aim and topic, imaging modality, and journal quartile. The 4 studies not based on the oncologic topic received significantly lower scores than the others (*p* = 0.01). Conversely, no statistically significant differences were found between papers according to first author category (*p* = 0.75), study aim (*p* = 0.9), and imaging modality (*p* = 0.48). Moreover, in studies published in first quartiles journals, the total RQS percentage was higher than that of investigations published in lower quartiles journals (median 19.4 vs. 8.3), but this difference was not statistically significant (*p* = 0.09).

**Table 3:** Subgroup analysis according to first author category, study aim, topic and design, imaging modality, and journal quartile

Group	Studies (*n*)	RQS percentage	*p* value
First author category			0.75
Medical	49	19.4 (0–30.6)	
Other	14	13.9 (3.5–29.9)	
Study aim			0.9
Diagnostic	44	18 (0–31.2)	
Prognostic	20	13.9 (2.1–30.6)	
Study topic			0.01
Oncology	59	19.4 (2.8–30.6)	
Other	4	0 (0–0)	
Study design			0.08
Single-center	52	12.5 (0–30.6)	
Multi-center	11	30.6 (15.3–33.3)	
Imaging modality			0.48
CT	27	22.2 (2.8–31.9)	
MRI	22	18.1 (0–30.6)	
PET	1	27.8	
US	14	5.6 (0–23.6)	
Journal quartile			0.09
First	31	19.4 (5.6–30.6)	
Others	29	8.3 (0–28.5)	

Values are expressed as number or median (interquartile range)

*RQS* indicates radiomics quality score [13]

## Discussion

Several radiomics-based investigations have been performed either with a diagnostic or prognostic aim for various ovarian pathologies, being especially focused on oncologic topics [9, 10, 17, 21–23]. Of note, CT was the most used diagnostic technique, even if it does not represent the imaging of choice in clinical routine.

However, despite the promising results, their translation to clinical routine still appears as a distant goal. This is particularly due to the complexity of the method and the low reproducibility of the several processes involved [3, 24, 25].

In our systematic review, the overall methodological rigor of ovarian radiomics investigations either with CT, MRI, PET, or US resulted to be unsatisfactory, with a median RQS total score of 6, corresponding to 16.7% of the maximum possible rating. Moreover, our results do not represent an exception. Indeed, previous studies highlighted that the overall methodological quality of radiomics studies is heterogenous and lower than desirable in various fields of medical imaging [14–16, 26–29]. In particular, Granzier et al for breast cancer, Ugga et al for meningioma, and Ursprung et al for renal cell carcinoma reported in their systematic reviews low average or median total RQS percentage, respectively of 11.8%, 19%, and 9.4% [15, 16, 27]. The trend of RQS over the years is fairly inconsistent, even though the increase in 2021 could represent a positive sign for the future. Considering that almost half of the investigations (31/63) were published in 2021, and that “how to” guides have been recently published aiming to standardize practice in radiomics, we could be cautiously optimistic that the tendency will be towards an overall improvement [2, 30]. A greater focus on this issue by journals and editors could also assist in improving the quality and diagnostic efficacy level of these types of investigations, in turn facilitating their introduction into clinical practice [21, 31].

Our systematic review has pointed out several issues in the included studies that will necessarily have to be solved in future radiomics-based research in the field of ovarian imaging. In detail, a comprehensive documentation of the imaging protocol is still lacking in some investigations; however, the corresponding item seems to have been better scored compared to the studies focused on different topics [14, 15]. Another major issue is represented by the overall lack of testing features robustness either to segmentation, scanner, or temporal variability. This could be at least partly due to the predominant retrospective design of the included investigations, which also represents a significant limitation. Segmentation definitely represents a crucial step in radiomics workflow as data are extracted from the segmented regions of interest. Of note, in the included papers, regions of interest were mostly annotated manually on medical images. However, the “ideal” segmentation strategy is still debated [32]. Some authors employ manual segmentation by expert readers as the ground truth, but this method can be highly time-consuming [33]. Automatic segmentation of the whole volume of interest could overcome this issue, but intensive user correction might be necessary for inhomogeneous lesions [34].

Moreover, as patient numbers are limited and countless radiomics features can be extracted, it is fundamental to reduce feature number, especially removing those poorly reproducible that could affect algorithm performance [3, 25, 35]. On a positive note, 76% of the reviewed papers performed feature reduction, thus lowering the risk of overfitting. Furthermore, even if the need of validating radiomics has been extensively discussed [36], less than half of the included investigations conducted a validation, either internal or external, of their results. However, the scores of this specific item obtained in the ovarian field are slightly better than those reported for prostate as well as breast cancer radiomics-based research [14, 15].

Open science remains a major issue also in ovarian setting, with 87% of the included papers not sharing their data and/or code. Publicly available datasets, such as the Cancer Genome Atlas Program and National Cancer Institute Imaging Data Commons, may represent a possible solution, helping to increase knowledge regarding the impact of varying factors in radiomics [37–39]. Of note, none of the included studies used public image protocols.

Subgroup analyses pointed out that the papers focused on the oncologic topic showed significantly higher RQS total scores than the non-oncologic ones. However, it should be taken into account that most of the studies (94%) aimed to assess radiomics performance in the field of oncology. Moreover, even if not reaching statistical significance, papers published in first quartile journals showed higher median RQS percentage than those published in the other quartile ones, possibly due to the greater demand of the high-ranking journals in terms of methodological rigor, especially regarding validation of the results.

Of note, the RQS may not represent the perfect tool to evaluate the methodological quality of a radiomics study. For example, due to the nature of its items, the RQS might penalize studies using deep learning algorithms, that are at risk of getting lower scores for lacking feature selection or multiple segmentations (which are not necessarily limitations in deep learning studies) [40]. Furthermore, the relative weight of some items might be unbalanced and penalize those preliminary, exploratory studies that were retrospectively designed but needed as a first ground on which stronger evidence must be built. Finally, it should be considered that generalizability is one of the key issues for the clinical translation of radiomics models but needs external independent validation that was rare in this experience (11%, 7/63). To increase the scientific merit and methodological robustness of radiomics studies, researchers might want to focus on validating previously published radiomics signatures using their datasets as independent validation cohorts rather than building new models. However, open science represents a necessary prerequisite to achieve this goal.

Our study suffers from some limitations that should be acknowledged. First of all, inter-reader agreement of RQS assessment was not explored; however, the two readers evaluating the papers had previous experience with this system of metrics [14, 28]. Second, since the field of radiomics is constantly evolving, even in terms of nomenclature, potential eligible investigations could have been missed. Finally, some included studies were published before the introduction of the RQS.

In conclusion, the overall scientific rigor of ovarian radiomics studies was unsatisfactory, resulting particularly lacking in terms of features reproducibility and formal validation of the results. More efforts towards a standardized methodology in the pipeline are needed to allow radiomics to become a viable tool for clinical decision-making.

## Supplementary Information

ESM 1 (DOCX 31 kb)

## Abbreviations

CT: Computed tomography
IQR: Interquartile range
MRI: Magnetic resonance imaging
PET: Positron emission tomography
PRISMA: Preferred Reporting Items for Systematic Reviews and Meta-analyses
PROSPERO: International Prospective Register of Systematic Reviews
RQS: Radiomics quality score
US: Ultrasound

## Declarations

### Guarantor

The scientific guarantor of this publication is Prof. Renato Cuocolo, MD, PhD.

### Statistics and biometry

No complex statistical methods were necessary for this paper.

### Methodology

• systematic review

• performed at multiple institutions
