# Supplementary material for: Ovarian imaging radiomics quality score assessment: an EuSoMII radiomics auditing group initiative
Source: Eur Radiol. 2022 Oct 27;33(3):2239–47. doi: 10.1007/s00330-022-09180-w (PMC9935717; doi:10.1007/s00330-022-09180-w)
Supplement: Supplementary file 1 — (DOCX 31 kb) [file 330_2022_9180_MOESM1_ESM.docx]

**Detailed Search String**

("radiomics” OR “texture” OR “histogram”) AND (“ovary” OR “ovarian”)

AND ("computed tomography" OR "CT” OR "magnetic resonance" OR “MRI” OR

“MR” OR “positron emission tomography” OR “PET” OR "ultrasound" OR "US")

**Supplementary Table. Characteristics of the included studies**

| **Author**  **(year)** | **Topic** | **First author**  **category** | **Study**  **aim** | **Imaging**  **modality** | **Journal quartile** | **Segmentation**  **method** | **Machine learning**  **algorithm** | **Population**  **(n)** |
| --- | --- | --- | --- | --- | --- | --- | --- | --- |
| Acharya (2013) | Oncology | Other | Diagnostic | US | I | Automatic, 2D | Decision Tree | 20 |
| Acharya (2014) | Oncology | Other | Diagnostic | US | III | Automatic, 3D | PNN, SVM, Decision Tree, KNN, Naive Bayes | 2600 |
| Ai (2021) | Oncology | Medical | Diagnostic | CT | III | Manual, 3D | LASSO, Ridge Regression, Logistic regression | 101 |
| Al-Karawi (2021) | Oncology | Medical | Diagnostic | US | II | Manual, 2D | SVM | 242 |
| An (2021) | Oncology | Medical | Diagnostic | CT | I | Manual, 2D | Random forest | 205 |
| Beer (2020) | Oncology | Medical | Diagnostic | CT | I | Manual, 3D | K-means | 20 |
| Chen (2021) | Oncology | Medical | Prognostic | CT | I | Manual, 3D | SVM, Logistic regression | 256 |
| Chen (2021) | Oncology | Medical | Diagnostic | CT | III | Manual, 3D | Random forest, Logistic regression | 256 |
| Chiappa (2021) | Oncology | Medical | Diagnostic | US | II | Manual, 2D | SVM | 274 |
| Chiappa (2021) | Oncology | Medical | Diagnostic | US | II | Manual, 3D | SVM, PCA | 241 |
| Danala (2017) | Oncology | Other | Prognostic | CT | I | Manual, 3D | NNS | 91 |
| Faschingbauer (2013) | Oncology | Medical | Diagnostic | US | I | Automatic, 2D | SVM | 105 |
| Fathi Kazerooni (2018) | Oncology | Other | Diagnostic | MRI | I | Manual, 3D | LDA | 67 |
| He (2020) | Oncology | Medical | Diagnostic | MRI | I | Manual, 3D | Logistic regression | 62 |
| Himoto (2019) | Oncology | Medical | Prognostic | CT | II | Manual, 3D | K-means, LASSO | 75 |
| Hu (2021) | Oncology | Medical | Diagnostic | CT | II | Manual, 3D | LASSO, Logistic regression | 110 |
| Jian (2021) | Oncology | Other | Diagnostic | MRI | I | Manual, 2D | LASSO | 294 |
| Khazendar (2015) | Oncology | Other | Diagnostic | US | NA | Manual, 2D | SVM | 187 |
| Kiruthika (2018) | Not-Oncology | Other | Diagnostic | US | III | Automatic, 2D | C-fuzzy clustering, K-means | 115 |
| Kyriazi (2011) | Oncology | Medical | Prognostic | MRI | I | Semi-Automatic, 2D | NA | 42 |
| Lee (2021) | Not-Oncology | Medical | Diagnostic | MRI | NA | Manual, 2D | NA | 25 |
| Li H (2021) | Oncology | Medical | Diagnostic | MRI | I | Manual, 3D | LASSO, Logistic regression | 217 |
| Li HM (2019) | Oncology | Medical | Prognostic | MRI | I | Manual, 3D | LASSO, SVM | 117 |
| Li HM (2020) | Oncology | Medical | Prognostic | MRI | II | Manual, 3D | NA | 56 |
| Li HM (2021) | Oncology | Medical | Diagnostic | MRI | II | Manual, 3D | NA | 126 |
| Li MR (2021) | Oncology | Medical | Diagnostic | CT | III | Manual, 3D | Logistic regression | 95 |
| Li NY (2021) | Oncology | Other | Diagnostic | MRI | III | Manual, 3D | Logistic regression | 46 |
| Li S (2021) | Oncology | Medical | Diagnostic | CT | I | Automatic, 2D | LASSO, Logistic regression | 134 |
| Li YA (2020) | Oncology | Medical | Diagnostic | MRI | I | Manual, 2D | LASSO, Logistic regression | 501 |
| Lu H (2019) | Oncology | Medical | Prognostic | CT | I | Manual, 3D | LASSO, K-means | 364 |
| Lu J (2021) | Oncology | Medical | Prognostic | MRI | I | Manual, 2D | Logistic regression | 70 |
| Lupean (2020) | Not-Oncology | Medical | Diagnostic | CT | IV | Semi-Automatic, 3D | Logistic regression | 43 |
| Lupean (2020) | Oncology | Medical | Diagnostic | MRI | II | Manual, 3D | NA | 28 |
| Meier (2019) | Oncology | Medical | Prognostic | CT | II | Manual, 3D | K-means | 88 |
| Mimura (2016) | Oncology | Medical | Diagnostic | MRI | II | Semi-Automatic, 3D | NA | 37 |
| Nero C (2020) | Oncology | Medical | Diagnostic | US | I | Manual, 2D | Logistic regression, SVM, XGBoost, Decision Tree, AutoML | 255 |
| Pan (2020) | Oncology | Medical | Diagnostic | CT | III | Manual, 3D | Logistic regression, LASSO | 103 |
| Park H (2021) | Oncology | Medical | Diagnostic | CT | II | Manual, 3D | Random forest, SVM, Logistic regression | 427 |
| Qi (2021) | Oncology | Medical | Diagnostic | US | III | Manual, 2D | Logistic regression | 173 |
| Qian (2020) | Oncology | Medical | Diagnostic | MRI | I | Manual, 3D | Logistic regression, LASSO | 61 |
| Rizzo (2018) | Oncology | Medical | Prognostic | CT | I | Manual, 3D | Iterative Hierarchical Clustering, Logistic regression | 101 |
| Seo (2021) | Not-Oncology | Medical | Diagnostic | CT | II | Manual, 3D | Decision Tree | 135 |
| Song (2021) | Oncology | Other | Diagnostic | MRI | I | Manual, 3D | LASSO, Logistic regression | 89 |
| Song (2021) | Oncology | Other | Diagnostic | MRI | I | Manual, 3D | Logistic regression | 104 |
| Stefan (2021) | Oncology | Medical | Diagnostic | US | II | Semi-Automatic, 3D | CNN, Multivariate regression | 56 |
| Ștefan (2021) | Oncology | Medical | Diagnostic | US | IV | Semi-Automatic, 3D | KNN | 120 |
| Vargas (2017) | Oncology | Medical | Prognostic | CT | I | Manual, 3D | Decision Tree, LASSO | 38 |
| Veeraraghavan (2020) | Oncology | Medical | Prognostic | CT | I | Manual, 3D | SVM | 75 |
| Wang R (2021) | Oncology | Medical | Diagnostic | MRI | I | Manual | CNN | 545 |
| Wang S (2019) | Oncology | Medical | Prognostic | CT/PET | III | Semi-Automatic, 3D | LASSO | 340 |
| Wang X (2021) | Oncology | Other | Prognostic | CT | I | Semi-Automatic, 2D | CNN | 44 |
| Wei C (2020) | Oncology | Medical | Diagnostic | MRI | I | Manual, 3D | Logistic regression | 53 |
| Wei W (2018) | Oncology | Other | Prognostic | CT | III | Manual, 3D | LASSO | 142 |
| Wei W (2019) | Oncology | Other | Prognostic | CT | NA | Manual, 3D | LASSO, Logistic regression | 94 |
| Yao (2021) | Oncology | Medical | Prognostic | US | II | Manual, 3D | LASSO | 111 |
| Ye (2021) | Oncology | Medical | Diagnostic | MRI | I | Manual, 3D | Random Forest, Logistic regression | 88 |
| Yi (2021) | Oncology | Medical | Prognostic | CT | I | Manual, 3D | LASSO, SVM, Random Forest | 178 |
| Yu XP (2021) | Oncology | Medical | Diagnostic | CT | III | Manual, 3D | LASSO, SVM | 182 |
| Yu XY (2021) | Oncology | Medical | Diagnostic | MRI | III | Manual, 3D | LASSO, Logistic regression | 86 |
| Zargari (2018) | Oncology | Other | Prognostic | CT | I | Semi-Automatic, 3D | GLM | 120 |
| Zhang H (2019) | Oncology | Medical | Diagnostic/  Prognostic | MRI | I | Manual, 2D | SVM, LASSO | 280 |
| Zhang L (2019) | Oncology | Medical | Diagnostic | US | I | Automatic | VGNet, AlexNet, GoogleLeNet, Fourier CNN, CNN, Random Forest | 428 |
| Zhu (2021) | Oncology | Medical | Diagnostic | CT | III | Manual, 3D | LASSO, Elastic Net, Logistic regression | 101 |

PNN, Probabilistic Neural Network; SVM, Support Vector Machine; KNN, K-Nearest Neighbor; PCA, Principal Component Analysis; NNS, Nearest Neighbor Search; LDA, Linear Discriminant Analysis; NA, not applicable; XGBoost, eXtreme Gradient Boosting; CNN, Convoluted Neural Networks; GLM, Generalized Linear Model; US, ultrasound; CT, computed tomography; MRI, magnetic resonance imaging.
